# Prescription opioid dispensing in Canada: an update on recent developments to 2018

**DOI:** 10.1186/s40545-020-00271-x

**Published:** 2020-10-23

**Authors:** Wayne Jones, Lenka Vojtila, Paul Kurdyak, Benedikt Fischer

**Affiliations:** 1 Centre for Applied Research in Mental Health and Addiction (CARMHA), Faculty of Health Sciences, Simon Fraser University, Vancouver, Canada; 2 Centre for Addiction and Mental Health, 33 Russell Street, Toronto, Ontario Canada; 3 Department of Psychiatry, University of Toronto, 250 College Street, 8th floor, Toronto, Ontario Canada; 4 Institute for Clinical Evaluative Sciences (ICES), 2075 Bayview Ave, Toronto, Ontario Canada; 5 Schools of Population Health and Pharmacy, Faculty of Medical and Health Sciences, University of Auckland, Auckland, New Zealand; 6 Department of Psychiatry, Federal University of São Paulo (UNIFESP), R. Sena Madureira, 1500 - Vila Clementino, São Paulo, Brazil

**Keywords:** Canada, Dispensing, Interventions, Opioids, Public health

## Abstract

Canada has been home to comparatively extreme developments in prescription opioid (PO) availability and related harms (e.g. morbidity, mortality) post-2000. Following persistent pan-Canadian increases in PO use, select control measures were implemented and PO dispensing levels—while only inconsistently by province—inverted, and began to plateau or decrease post-2012. We examined annual PO dispensing levels in Canada up until 2018, based on representative prescription sample data from community-based retail pharmacies. Annual prescription-based dispensing data were converted into defined daily doses/1000 population/day by province, and mainly categorized into ‘weak’ and ‘strong’ opioids. All provinces indicated decreasing trends in strong PO levels in most recent years, yet with inter-provincial differences of up to one magnitude in 2018; in about half the provinces, dispensing fell to below-2005 levels. British Columbia had the largest decline in strong PO dispensing from its peak rate (− 48.5%) in 2011. Weak opioid dispensing trends remained more inconsistent and bifurcated across Canada. The distinct effects of individual—including many provincially initiated and governed—PO control measures urgently need to be evaluated. In the meantime, recent reductions in general PO availability across Canada appear to have contributed to shortages in opioid supply for existent, sizable (including non-medical) user populations and may have contributed to recent marked increases in illicit opioid use and harms (including rising deaths).

## Introduction

In North America, the epidemiological picture of high availability and use of prescription opioids (POs), and related public health consequences, are well-documented [1–4]. Post-2000, PO dispensing began to rise rapidly, resulting in the USA and Canada becoming the world's two countries with the highest PO use rates [5]. In parallel, key PO-related adverse consequences—including increases in non-medical use, morbidity (e.g. hospitalizations) and mortality (e.g. fatal poisonings)—unfolded, leading to a PO-related ‘public health crisis’ including unprecedented reductions in life expectancy [1, 3, 6]. Most of the above population-level morbidity and mortality outcomes have been shown to be strongly correlated with PO dispensing volumes [7–9].

North American governments and regulators began to more actively implement measures to address increasing PO-related harms post-2010 [10–13]. In Canada, this has included a variety of interventions—some fragmented given that key aspects of health policy or regulations have provincial (vs. national) oversight. Measures have included the delisting of slow-release oxycodone (‘Oxycontin’) from provincial formularies (in 2012), newly introduced and/or strengthened (provincial) prescription monitoring programmes combined with enhanced prescriber education as well as select disciplinary action against over-prescribers and introduction of restrictive opioid prescribing guidelines (in 2016/2017) [10, 14, 15]. In addition there has been growing public discussion and awareness on the consequences of opioid use over this period [16, 17].

While PO dispensing peaked in the US in about 2010 and subsequently declined by about 25% in response to control measures implemented, it remains globally unsurpassed for its opioid consumption levels [18–20]. In Canada, similar developments have lagged and have been less consistent, with select decreases in PO dispensing identified for half of the provinces post-2012 [21, 22]. Throughout this period, PO dispensing levels across Canadian provinces have been quite diverse, including up to threefold variations in prescribing volumes [21, 23]. Despite these overall reductions in dispensing, increases in key opioid-related harms (e.g. mortality, morbidity) have occurred across North America in recent years, increasingly driven by expanding involvement of illicit, highly toxic opioid products in the past five years [24–27].

In the following and extending previous work, we briefly examine and update on trends and patterns in PO dispensing in Canada for the period up to 2018, with primary focus on recent developments as well as population health implications.

## Methods

Present analyses are based on annual PO retail dispensing data across Canada (specifically, the ten Canadian provinces) from 2005 to 2018. Raw aggregated update data were obtained from the IQVIA’s CompuScript database, which monitors prescription-based retail transactions for medications, and had been used for previous related analyses [28–31]. Totals for the number of PO prescriptions and number of units dispensed for each opioid product were provided by IQVIA aggregated by region (provinces), opioid molecule (codeine, fentanyl, hydrocodone, hydromorphone, meperidine, methadone, morphine, oxycodone or tramadol), product names, form (solid, liquid, etc.) and strength. Methadone formulations were provided but excluded from the analyses since they are primarily used for addiction treatment, and dispensing modes greatly vary [32]. As tramadol has only been available in Canada since 2006 data were not available for the full study period. However, their inclusion (among ‘weak opioids’) showed to not alter observed overall patterns within provinces [33].

The individual provincial summary of total dispensing data were converted to annual defined daily doses per 1000 population per day (DDD/1000/day) values. DDD estimates were obtained from the World Health Organization’s (WHO) Anatomical Therapeutic Chemical (ATC) classification and DDD measurement methodology, population statistics calculated from the Statistics Canada’s CANSIM table 051-0005 [34–36]. Based on the WHO’s pain ladder, tramadol and codeine formulation products were defined as ‘weak’ opioids, all other formulations were combined into ‘strong’ opioids for descriptive analysis [37].

**Fig. 1:**
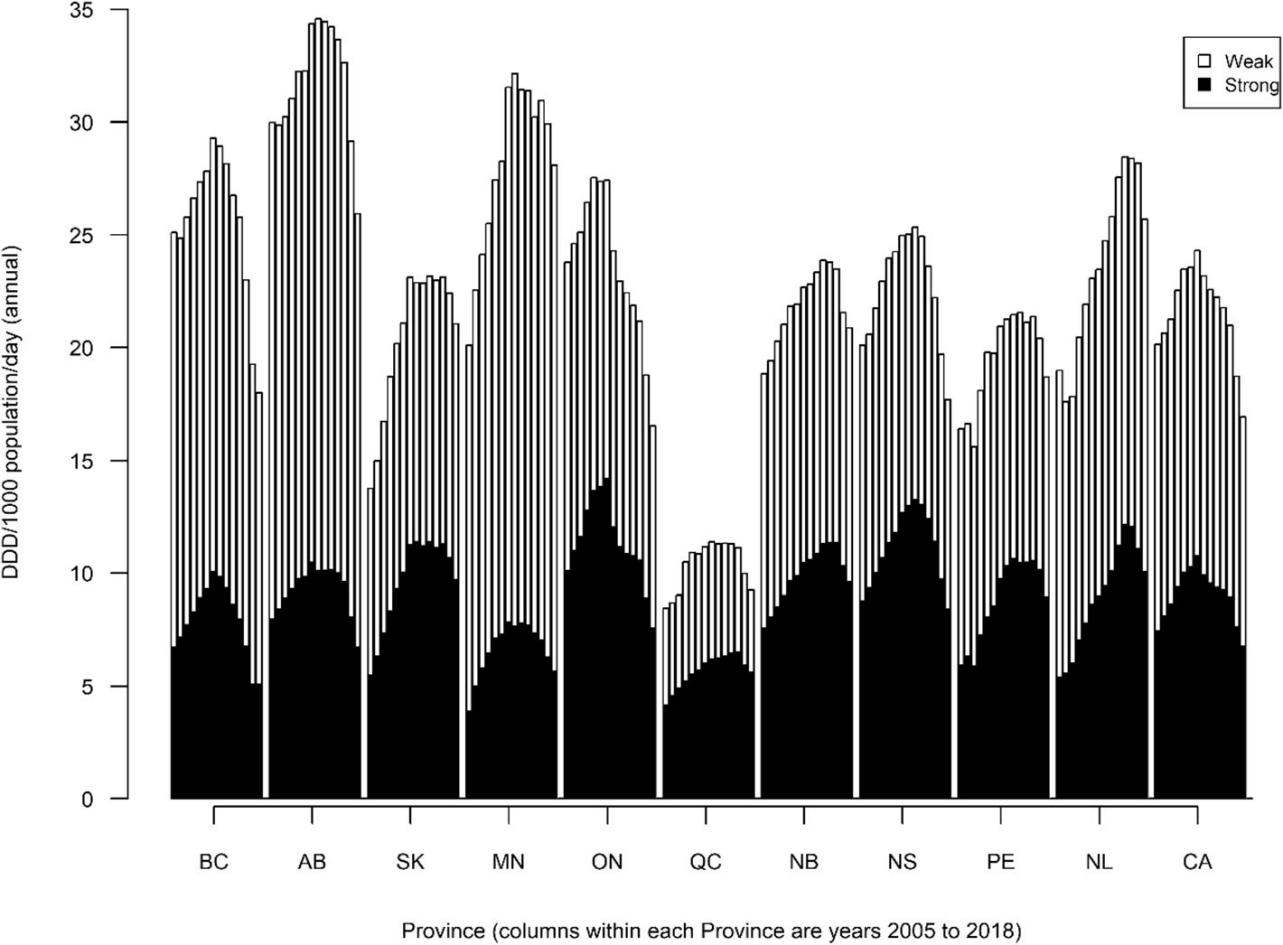
Total prescription opioid dispensing (without methadone) in annual DDD/1000 population/day in Canada, by province, 2005–2018. Full names of provinces (acronyms used in text and figure): BC, British Columbia; AB, Alberta; SK, Saskatchewan; MN, Manitoba; ON, Ontario; NB, New Brunswick; NS, Nova Scotia; PE, Prince Edward Island; NL, Newfoundland and Labrador; CA, Canada (total)

Descriptive analyses for the different PO categories included low- and high-ranking values in dispensing and change rates, by province and over-time [Fig. 1]. No ethical approval was required for the present study based on the nature of the non-personalized, population-aggregate dispensing data used for analyses.

## Results

In 2018, British Columbia (BC) (5.1 DDD/1000 pop/day) featured the lowest strong PO dispensing rate and Newfoundland and Labrador (NL) had the highest (10.1 DDD/1000 pop/day), approximately double the rate. Most provinces reported their peak rates for strong PO dispensing in 2011 but no later than 2016. All provinces indicated multi-year decreasing trends in strong PO dispensing in recent years and lower dispensing compared to previous peak rates; four out of the ten provinces indicated strong PO dispensing rates in 2018 that were below 2005-levels. Compared with respective peak years, BC had the greatest (− 49.5% from 2011), Quebec the smallest (− 13.8% from 2016) relative reduction in strong PO dispensing within the study period.

Correspondingly, BC (2.6 DDD/1000 pop/day) featured the lowest weak PO dispensing rate and Alberta (AB) had the highest (17.1 DDD/1000 pop/day) in 2018, equating to more than a sixfold difference. All provinces had lower dispensing rates for weak POs compared to previous peak rates observed during the study period, and all but one of the provinces (Saskatchewan [SK]) had weak PO dispensing rates that were below 2005 levels. Compared with respective peak years, ON had the greatest (− 43.1% from 2005) and NL the smallest relative reduction (− 11.8% from 2014) in weak PO dispensing within the study period.

## Discussion

The PO dispensing data presented is drawn from a stratified, representative sample of about 6,000 (approximately two thirds of the total) retail pharmacies across Canada, including a continuously refreshed sub-sample providing the pharmaceutical dispensing data comprising the large majority of prescriptions at the national level [28, 38]. The Compuscript panel projects the sample-based prescription data to the universe of pharmacies by province, with a sampling error that is estimated to be low (5–10%) and data representativeness considered good. About 80% of the total of POs in Canada are dispensed through retail pharmacies (with other routes, including hospital-based dispensing, not captured) [29]. Over this time period, Canadian pharmacies could sell select codeine (e.g., cough or mild pain/fever) medication) 'over-the-counter' without a prescription; however, these sales are not included in the present data [39, 40]. Overall, this is probably the best available data snapshot on country-wide PO consumption based on DDD values, interpreted as a drug’s average maintenance dose per day for its main indication for an average adult. DDDs are a measurement unit with limits in accuracy, yet are seen as superior to indicators like crude prescription numbers, especially for comparative analyses [41–43].

Our data—building on previous pharmaco-epidemiologic analyses—showed that population-level PO dispensing has undergone distinctly oscillating waves and changes in Canada during the period 2005–2018 [22, 23, 29]. While there were stark pan-Canadian increases especially in strong PO dispensing up until 2011, making Canada a global co-lead in opioid usage, all provinces have featured subsequent declines in dispensing more recently [5, 20]. These recent declines in strong PO dispensing, however, vary considerably in terms of timing and relative reductions [21, 22]. Nevertheless, it is a fair overall summary that following a general ‘wave’ of strong PO dispensing increases until halfway through the study period, and an inversion occurred involving a second, ‘downward’ wave in strong PO dispensing thereafter in Canada.

Notably, by 2018, some provinces have reduced their strong PO dispensing to below 2005 levels—a time when more generous opioid dispensing was actively promoted towards improved chronic pain care outcomes [44–46]. While the prevalence of chronic pain in the Canadian population does not appear to have changed, preeminent pain treatment guidelines and other prescription-related advise have undergone a virtual ‘180 degree’ turn, basically from recommending ‘generous opioids provision’ to ‘sparing or “last resort” resort use only’. One would expect tangible empirical impact on pain care outcomes from a period in which opioid usage inverted by such magnitudes in a national population over the period of less than two decades [15, 47–49]. Moreover, dispensing trends for weak opioids showed less consistent patterns in an environment increasingly cautious about medical codeine usage [50]: about half the provinces featured substantial overall declines, others remained generally stable in weak PO dispensing, while at differential quantity levels.

It is reasonable to assume that the pronounced recent decreases especially in strong PO dispensing across Canada are an overall consequence of the various opioid control measures—including opioid product delisting, prescription monitoring and revised prescription guidelines—implemented in Canada over the past decade [49, 51–53]. Unfortunately, only most limited evaluations exist as to the impact of these individual interventions on variations in PO availability—whether on provincial or national levels—even though this information would be crucially important for evidence-based interventions and policy development towards optimized opioid control and related benefits and/or harms [2, 10, 54]. Such analyses urgently need to be conducted with appropriate, rigorous approaches, for example time-series and other analysis methods [52, 55]. Overall and despite recent declines combined with some inter-provincial alignment, the total of strong PO dispensing patterns remains rather heterogeneous across Canadian provinces, as further exemplified by variations in the types of opioid formulations used [21].

One would expect that the recent declines in population-level opioid dispensing in Canada co-occurred with parallel benefits in opioid-related public health outcomes. Unfortunately, this appears not to be the case, or reductions from excessive prescribing levels occurred too late, evidence for improvements in pain care outcomes is lacking, and opioid-related mortality and morbidity indicator outcomes have substantially risen further in recent years (e.g. 2017/18) [56–58]. Contributing factors to this dire situation are multi-layered and include the increasing availability and non-medical use—despite substantially expanded public health and treatment interventions—of illicit, highly potent and toxic opioid products (e.g. fentanyl or analogues) by many at-risk users in locations across Canada [25, 26, 59]. While these developments in rising illicit opioid use and harms appear to be extrinsically driven, the impact of a suddenly and rapidly shrinking (direct or indirect/diverted) medical opioid supply for large populations habituated into (medical or non-medical) opioid use in Canada need to be better analyzed and understood [60, 61]. Further epidemiological analyses and improved intervention and policy development for this opioid-related public health crisis are urgently required.

## Conclusion

Following persistent increases that gave Canada one of the highest opioid use rates in the world, an assortment of various policy interventions implemented post-2012 to curb general opioid availability and adverse consequences in the population were introduced, and prescription opioid dispensing showed variably decreasing trends in each of ten provinces. Both the magnitude of the decreases and current opioid dispensing levels continue to be rather heterogeneous across the provinces and so raise questions about differential medical use and needs standards, as well as the effectiveness of policy measures for opioids. From a public health perspective, the substantive decreases in medical opioid availability appears to have led to unintended consequences of supply shortages for non-medical opioid use resulting in extensive adverse outcomes (e.g. overdose fatalities) related to increases in illegal opioid (e.g. fentanyl and analogues) availability and use.

## Data Availability

The datasets (Statistics Canada) analysed during the current study are publicly available from Statistics Canada [https://www150.statcan.gc.ca/t1/tbl1/en/tv.action?pid=1710000901]; the opioid dispensing data are not publicly available since underlying data were commercially obtained and used under licence. The commercial data provider had no involvement in or influence on analyses, interpretation or publication of the data.

## Abbreviations

PO: Prescription opioid(s)
